# Regenerating and repairing degenerative intervertebral discs by regulating the micro/nano environment of degenerative bony endplates based on low-tension mechanics

**DOI:** 10.1186/s12891-022-05422-6

**Published:** 2022-05-16

**Authors:** Yan-Jun Che, Jiang-Bo Guo, Yue Feng Hao, Zong-Ping Luo

**Affiliations:** 1 Orthopedics and Sports Medicine Center, The Affiliated Suzhou Hospital of Nanjing Medical University, Suzhou, 215008 Jiangsu Province China; 2 Department of Orthopaedics, Orthopaedic Institute, The First Affiliated Hospital of SooChow University, 708 Renmin Rd, SuZhou, Jiangsu 215007 People’s Republic of China

**Keywords:** Intervertebral disc degeneration, Biomechanics, Low tension traction (LTT), High tension traction (HTT), Regeneration, Repairing

## Abstract

**Background:**

Conservative treatment is the recommended first-line treatment for degenerative disc diseases. Traction therapy has historically been one of the most common clinical methods to address this, but the clinical effect remains controversial.

**Methods:**

Forty-two six-month-old male Sprague-Dawley rats were randomly divided into six groups: the model group (Group A, four coccyx vertebrae (Co7-Co10) were fixed with customized external fixators, and the vertebral disc degeneration model was constructed by axial compression of the target segment Co8 - Co9 for 4 weeks), the experimental control group (Group B, after successful modeling, the external fixation device was removed and self-rehabilitation was performed) and four intervention groups (Groups C to F): Groups C and E: Co8 - Co9 vertebrae compressed for 4 weeks followed by two or 4 weeks of high tension traction (HTT), respectively, and Groups D and F: vertebrae compressed for 4 weeks followed by two or 4 weeks of low-tension traction (LTT), respectively. Imaging tests (X-ray and MRI) were performed to assess disc height and T2 signal intensity at each time point. After the experiment, the animals were euthanized, and the caudal vertebrae were collected for analysis of intervertebral disc histopathology, proteoglycan content, and micronanostructure of the annulus fibrosus, nucleus pulposus and bony endplate.

**Results:**

Signs of tissue regeneration were apparent in all four intervention groups. After two to 4 weeks of intervention (HTT and LTT), the morphology of pores in the bony endplate, their number, and diameter had recovered significantly compared with those in Group A. The LTT group was superior to the HTT group, and the 4w in situ group was significantly superior to the 2w group. Meanwhile, the histological scores of discs, the mean fibril diameter and modulus of annulus fibrosus were significantly improved compared with the control groups, and the LTT group was superior to HTT group.

**Conclusions:**

Low-tension traction better promotes active reconstruction of bony endplates and improves the elastic modulus and micro/nanostructure of the disc. Thus, it further promotes the regeneration and repair of intervertebral discs.

## Introduction

As a special closed biomechanical structure, the intervertebral disc (IVD) is composed of many interacting and related tissues and is the largest avascular and noninnervated tissue in the body [1]. The supply of nutrients and the transport of metabolites are carried out principally via exchange through the end plate in the form of molecular dispersion fluid [2], which also results in the internal environment being consequently affected by the nutritional environment, mechanical distribution, cytokines, and components of the extracellular matrix (ECM) [3]. The complete endplate (EP) system is composed of the bony endplate (BEP) and its overcast cartilage endplate (CEP). For intervertebral disc degeneration (IVDD), CEP has been the focus of clinical and scientific attention. CEP is not prone to hardening or calcification due to changes in the mechanical environment [4], and BEP, serves as a mechanical barrier and nutritional channel between the CEP and vertebral bone [5]. However, the role of BEP in IVDD is still controversial [6]. Literature reports and our previous studies have revealed that the degeneration and disease of BEP, as the supporting structure of CEP and the anchor part of the annulus fibrosus (AF), will directly weaken the mechanical properties of AF and further change the mechanical stress distribution in the IVD, resulting in IVDD [6–8]. In addition, several potential pathogenic factors may also lead to IVDD through EP [2, 7]. Studies by Splendiani et al. have shown that disc changes can be observed with dynamic MRI (supine to standing), which revealed the correlation between “biomechanical stress” and “active disc disease” of low back pain. Additionally, the relationship between degenerative disc and endplate alterations, lumbar weight bearing and pain was further elucidated, indicating that EP, especially BEP, is instrumental in the process of disc biomechanics and degeneration [9]. At present, conservative treatment remains the recommended first-line treatment for cervical pain, shoulder pain and lower back pain (LBP) caused by disc degeneration [10, 11], but the clinical effect is controversial. In clinical practice, the disc from the stress source is often repaired by improving its abnormal stress environment (such as traction therapy), but in the absence of information on the biomechanical environment of BEP, the microenvironment of the disc is most likely not substantially improved. Therefore, clarifying the mechanical microenvironment of BEP is also key in restarting the degenerative disc repair cascade. Based on our previous research work, this study used low-tension traction to reshape the degenerative endplate microenvironment to repair or reverse the degenerative intervertebral discs to a certain extent.

## Materials and methods

### Animal models and experimental groups (Table 1 and Fig. 1)

In this study, 42 six-month-old male Sprague-Dawley rats (weight [mean ± standard deviation]: 435 ± 15 g) were used. Animal experiments were approved by the Institutional Animal Care Committee of the Laboratory Animal at Nanjing Medical University, Jiangsu Province, China. Rats were randomly assigned to one of six groups (*n* = 7 in each group, Table 1). Group A: Model group (caudal vertebrae were immobilized using a bespoke external device to fix four caudal vertebrae (Co7 - Co10), while Co8 - Co9 vertebrae underwent 4 weeks of compression to induce disc degeneration); Group B: experimental control group (device was removed after the 4 weeks of compression described in Group A). The remaining four groups represented intervention groups (Groups C and E: Co8 - Co9 vertebrae compressed for 4 weeks followed by two or 4 weeks of excessive traction (high tension traction, HTT), respectively; Groups D and F: vertebrae compressed for 4 weeks followed by two or 4 weeks of in situ traction (low-tension traction, LTT), respectively. X-ray and magnetic resonance imaging (MRI) were performed at each time point to measure disc height and T2 signal intensity. Finally, the animals were euthanized, and tail vertebrae were harvested for analysis of intervertebral disc histopathology, proteoglycan content, elastic modulus of fibers of the annulus fibrosus (AF), nucleus pulposus (NP) and microstructure of the bony endplate.

**Table 1:** Summary of study design

Group	Category	Instrumented level (Co8 - Co9)	No. of Animals
A	Model	Com - 4 weeks	7
B	Exp-control	Com - 4 weeks (remove devices)	7
C	Int	HTT - 2w(*High tension traction - 2 w*)	7
D	Int	LTT - 2w(*Low tension traction - 2 w*)	7
E	Int	HTT - 4w(*High tension traction - 4 w*)	7
F	Int	LTT - 4w(*Low tension traction - 4 w*)	7

*Com* Compression, *Int* Intervention, *Exp – control* Experimental control, *HTT* High tension traction, *LTT* Low tension traction

**Fig. 1:**
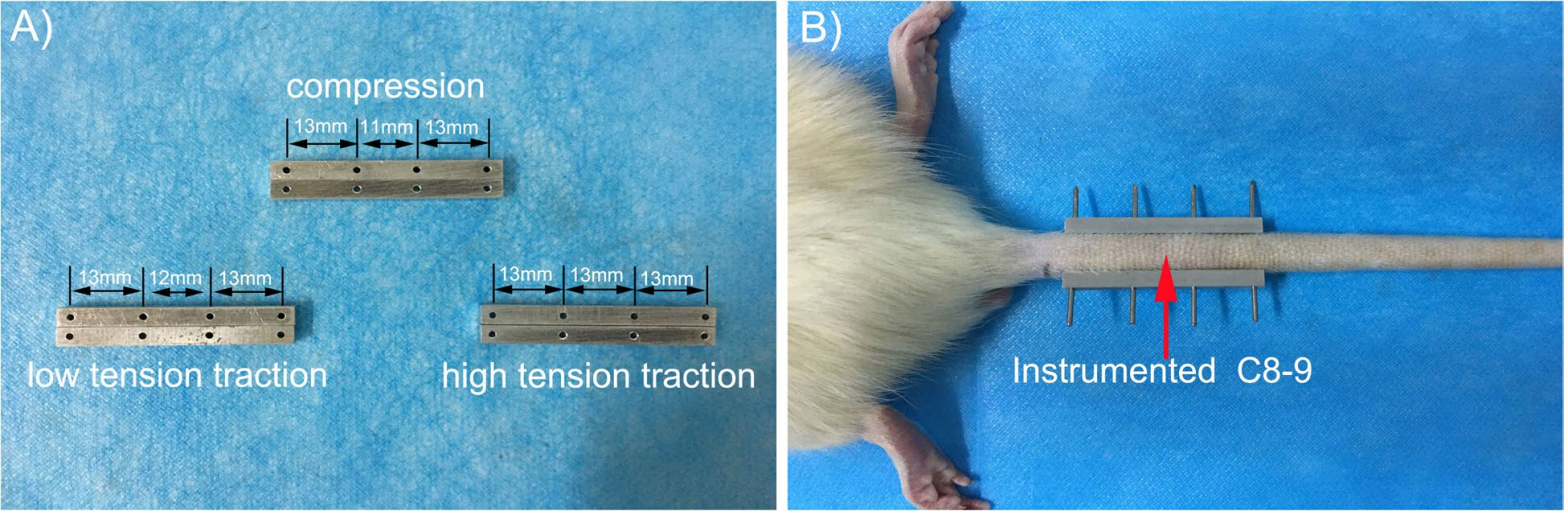
Animal model. **a**) Schematic diagram of the external coccyx device: model group (compression). The caudal vertebrae were immobilized using a custom-made external device to fix four caudal vertebrae (Co7-Co10). The hole spacing of Co8-Co9 was 11 mm, and both hole spacings of Co7-Co 8 and Co9-Co10 were 13 mm. Intervention groups (low tension traction): The hole spacing of Co8-Co9 was 12 mm, and both hole spacings of Co7-Co 8 and Co9-Co10 were 13 mm. Intervention groups (high tension traction): the hole spacings of Co7-Co8, Co8-Co9, and Co9-Co10 were all 13 mm. **b**) Coccyx external fixator fixation diagram, Co8 - Co9 for experimental observation of the intervertebral disc

### Disc height measurements and magnetic resonance imaging analysis

The caudal discs were evaluated and analyzed by X-ray and MRI imaging after each time point [7, 12, 13]. Intervertebral disc space was measured using the Masuda method [12]. MRI scanning was assessed by three MRI physicians and three spine surgeons using the Pfirrmann score [7, 14].

#### Histological analysis and measurement of glycosaminoglycan (GAG) content

At the end of the experiment, the target discs (Co8-Co9) were obtained for histopathological analysis. Disc tissue specimens were fixed, decalcified, sectioned and then stained prior to microscopic observation [7, 13, 15]. Histological evaluation was based on the grading system developed by Han et al. [13, 16]. Alcian blue tissue staining was used to quantify the proteoglycan content [17]. To determine the intervertebral disc proteoglycan content as accurately as possible, we evaluated the proteoglycan alcian blue staining level by referring to the above method [17, 18].

### Evaluation of the bony endplate by SEM

Based on our previous study [3, 7, 13, 18], the bony endplates of each group were observed and evaluated by scanning electron microscopy (SEM). In each group, the endplates of intervertebral discs Co8-Co9 were separated, digested by collagenase, and then dried in natural air and a critical point dryer. Finally, the palladium alloy was sprayed by an ion sputtering machine, and the specimens were observed by SEM.

### Atomic force microscopy (AFM) imaging and nanomechanical testing

**Fig. 2:**
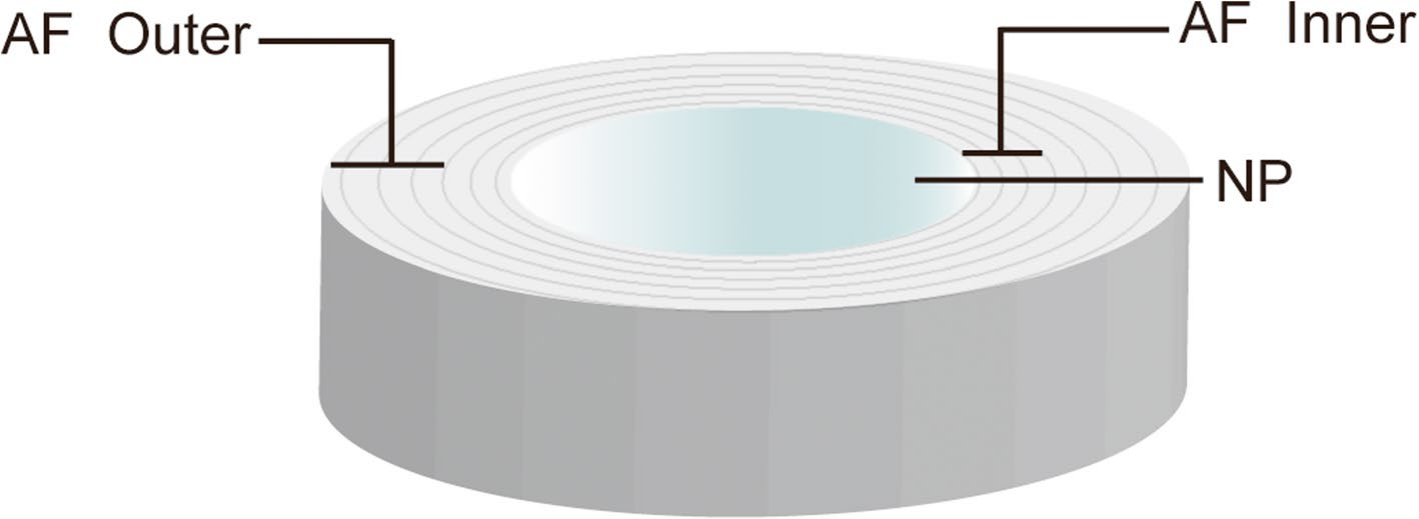
Schematic diagram of the different scanned regions in the disc using AFM

Atomic force microscopy (AFM) was used to measure the characteristics of the NP and AF collagen fibers, including their structure, morphology, and elastic moduli [7, 19]. AF samples were divided into two scanning regions (outer and inner annulus fibrosus, Fig. 2) based on previous studies [19].

### Statistical analysis

Data were managed and statistically analyzed using GraphPad 6 (GraphPad Software, USA) and SPSS 24.0 (IBM SPSS Inc., Chicago, IL, USA) software. The results are expressed as the means ± standard deviation (SD). The significance of differences between study groups was calculated using one-way analysis of variance (ANOVA) combined with a Holm-Sidak multiple comparison test to assess the influence of loads and duration. The level of statistical significance was set to 0.05.

## Results

All animals survived to the end of the experiment and were able to bear the use of external custom devices. During the experiment, one rat developed coccygeal needle tract infection, which was cured after timely treatment with local anti-inflammatory drugs without affecting the experimental results. Because of the simple operation of the external customized device and its good tolerance in rats, the use of the external fixation device used in the previous experiment [13] was continued in this study. However, the hole spacing of the external fixator in the low tension traction group was adjusted (Fig. 1a).

### Intervertebral disc height and T2 signal intensity

**Fig. 3:**
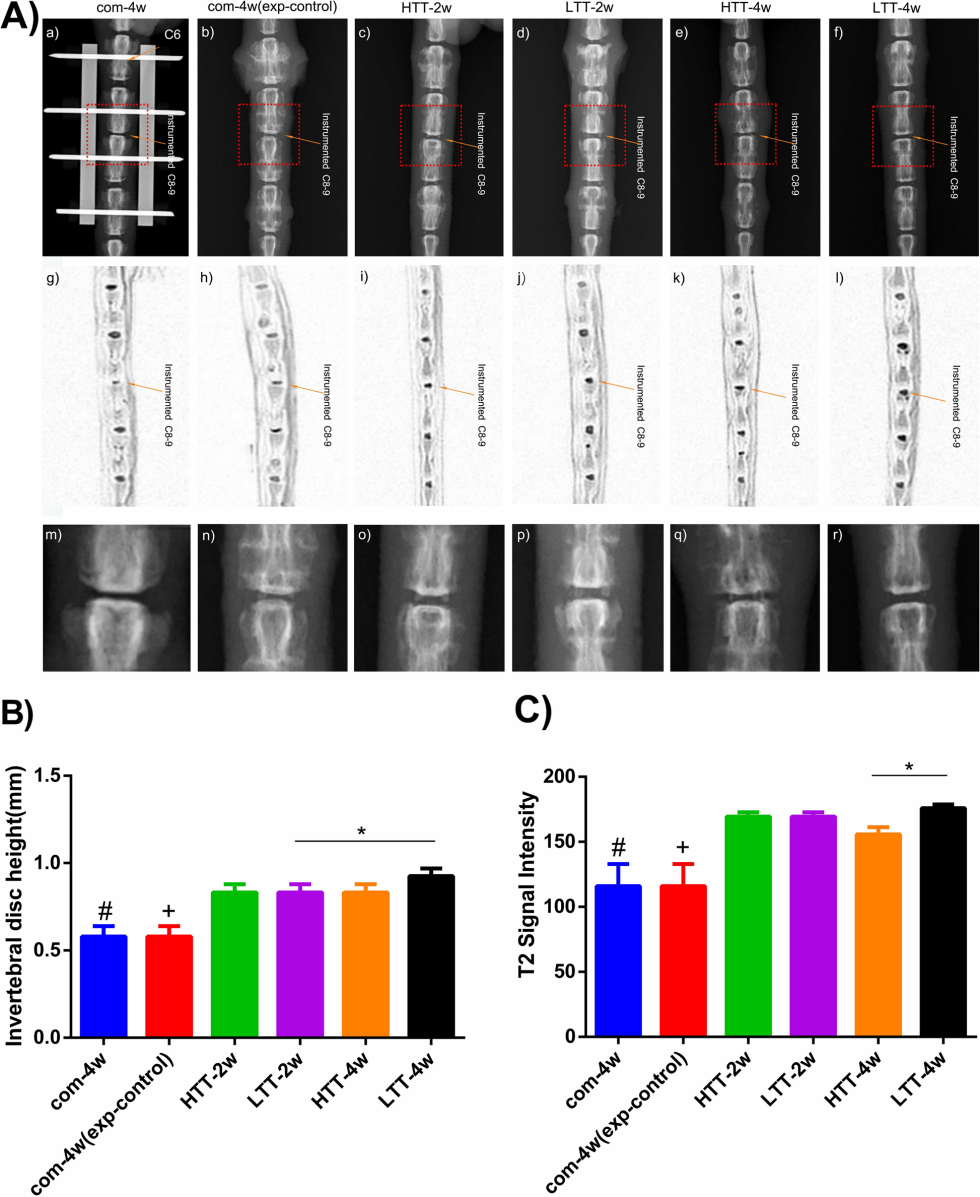
Intervertebral disc height and T2 signal intensity. **A**) Radiographs (a - f) were obtained under anesthesia using a digital, self-contained X-ray machine cabinet. MRI scans (g - l) are the inverse of MRI images, using a magnetic field to create images in which the size and hydration status of the NP can be ascertained, depending on T2 signal intensity. Fig (m-r) is a partial enlargement of the red dotted square box in the top line figure (a - f). **B-C**) Disc height (IDH) and T2 signal intensity: (#) indicates a significant difference compared with the intervention groups (Groups C to F, *p* < 0.0001); (+) indicates a significant difference compared with the intervention groups (Groups C to F, *p* < 0.001); and (*) indicates a significant difference between Groups D and F or Groups E and F (*p* < 0.05)

At the end of the experiment, after 4 weeks of axial compression, the intervertebral space and T2 signal intensity of the Co8-Co9 vertebrae in Groups A - F were significantly decreased (Fig. 3A). After two to 4 weeks of continuous traction (LTT and HTT), the Co8 − Co9 intervertebral space of rats in Groups C to F was significantly greater than that in Groups A and B (*p* < 0.05, Fig. 3B). In addition, the recovery of disc height in Groups B to E was lower than that in Group F (*p* < 0.05, Fig. 3B). However, no significant difference was detected between Group C and Group D or between Group E and Group F (*p* > 0.05). The recovery of T2 signal intensity of Co8 − Co9 intervertebral disc in the intervention Groups C to F was significantly greater than that in Group A (*p* < 0.05), although compared with Group A, Group B also recovered to a certain extent (*p* > 0.05), but was still lower than the four intervention groups (*p* < 0.05), and in particular, Group F vs Group E (*p* < 0.05, Fig. 3C). According to Fig. 3A (m-r), osteophyte formation of different degrees occurred around the disc, including the lateral edge of the adjacent vertebral body, simultaneously with disc height recovery in the intervention groups (Groups C to F), with different traction tensions and traction cycles. Osteophyte formation in the LTT - 2w and - 4w groups (Groups D and F) was less than in the HTT - 2w and - 4w groups (Groups C and E), and the LTT - 4w group was significantly better in terms of osteophyte formation than the LTT - 2w group. A large amount of stress osteophyte formation was observed in the HTT groups (Groups C and E) around the intervertebral disc and at the outer edge of the adjacent vertebrae. Although the intervertebral disc height in the control group (Group B) partially recovered, compared with that in the model group (Group A) during the self-recovery process after the removal of external fixation devices, a large number of compensatory osteophytes were still observed around the intervertebral disc and adjacent vertebral bodies, indicating that intervertebral instability factors remained present.

### Histological analysis and measurement of glycosaminoglycan (GAG) content

**Fig. 4:**
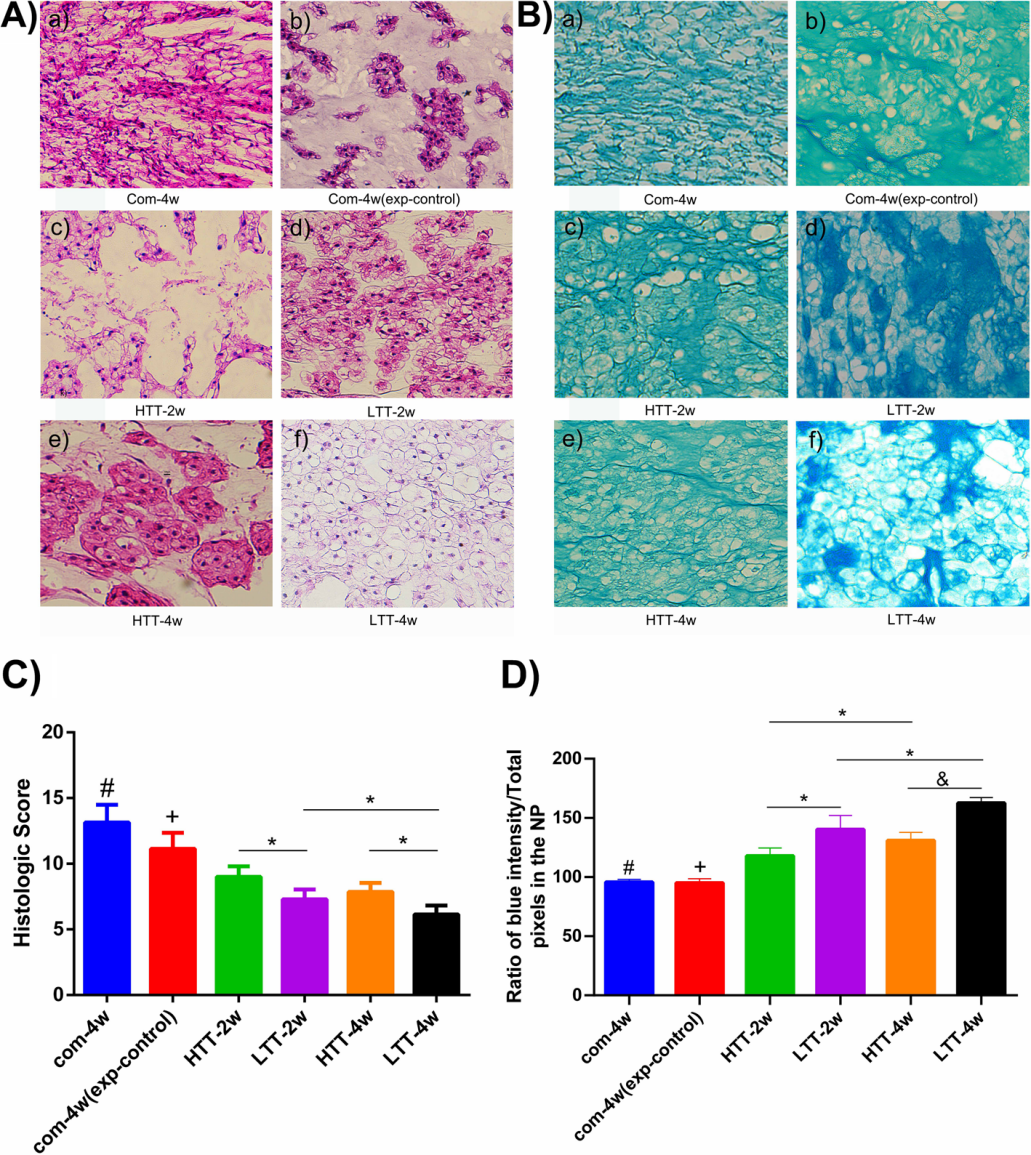
**A**) Hematoxylin-eosin tissue staining (H&E): a-f: IVD sections of each group were magnified 400 times. **B**) Alcian blue tissue staining: a-f: IVD sections of each group were magnified 400 times. **C**) Histological score: (#) indicates a significant difference compared with other groups (*p* < 0.05); (+) indicates a significant difference compared with other groups (*p* < 0.05); (*) indicates a significant difference between the two groups (*p* < 0.05). **D**) Proteoglycan assay: (#) indicates significant difference compared with other groups except Group B (*p* < 0.05); (+) indicates a significant difference compared with other groups except Group A (*p* < 0.05); (*) indicates a significant differences between the NP of the two groups were detected (*p* < 0.05); (&) indicates a significant differences between the two groups (*p* < 0.0001)

After 4 weeks of compression, NP showed typical degenerative morphological changes, along with a decrease in disc height, that is, a gradual decrease in NP cells and a gradual loss of proteoglycan content (Fig. 4A-B). The increase in local density and uneven distribution of NP cells led to the disturbance and destruction of the AF. The intervention groups (Groups C to F) showed signs of tissue regeneration after LTT and HTT-2-4 weeks (Fig. 4A-B). The fibrotic tissue of compensatory hyperplasia decreased in the four intervention groups due to progressive recovery of disc height and volume, but the recovery was poorer in the HTT-2-week group (Group C) than in the other three intervention groups (Fig. 4A-B). Compared with the model group (Group A), the LTT 4-week group (Group F) showed significantly less fibrotic tissue. Intervertebral disc NP cells were more evenly distributed in the LTT group (Groups D and F) than in the HTT group (Groups C and E). Additionally, the histological score of the intervention group (Groups C to F) was significantly lower than the corresponding score of the model group (Group A) and the experimental control group (Group B), namely, Group C vs Group D (*p* < 0.05, Fig. 4C), Group E vs Group F (*p* < 0.05) and Group D vs Group F (*p* < 0.05). Compared with the model group (Group A), the total proteoglycan content of the intervertebral disc NP in the intervention groups (Group C-F) significantly increased (*p* < 0.0001, Fig. 4D), namely, Group C vs Group D (*p* < 0.05), Group E vs Group F (*p* < 0.0001), and Group F vs Group D (*p* < 0.05) (*p* < 0.01). However, no significant difference in NP total proteoglycan content was detected between Group A and Group B (*p* > 0.05).

### Evaluation of the bony endplate

**Fig. 5:**
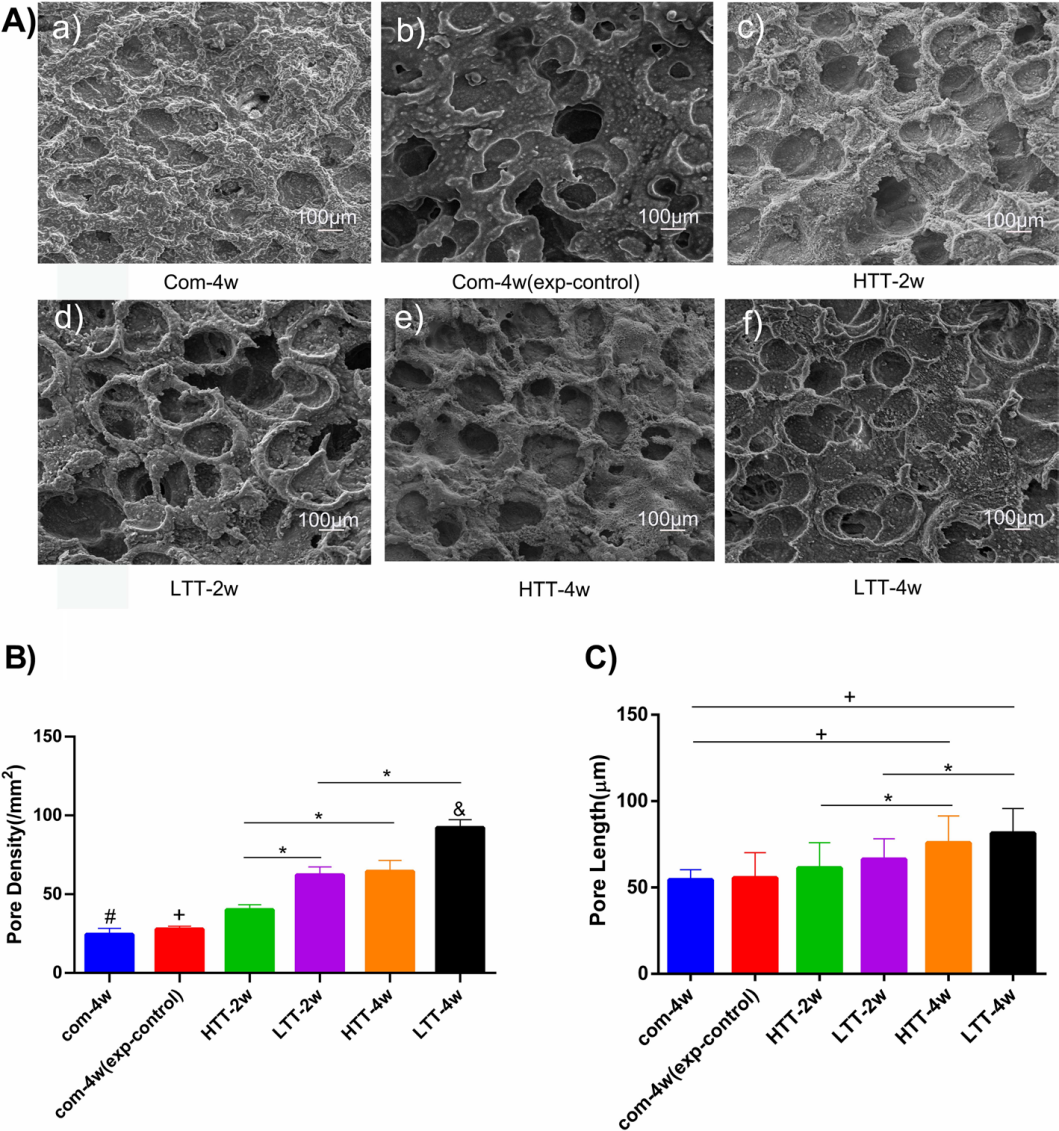
Bone endplates were evaluated by SEM. **A**) a-f: 1000x magnification for each end plate. **B**) Pore density: (#) indicates a significant difference compared with the other groups except Group B (*p* < 0.05). (+) represents a significant difference compared with the other groups except Group A (*p* < 0.05). (*) indicates statistical significance between the two groups (*p* < 0.05). (&) indicates significant differences in other groups (*p* < 0.0001). **C**) Pore diameter: (*) indicates statistical significance between the two groups (*p* < 0.05). (+) indicates a significant difference between the two groups (*p* < 0.0001)

The bony endplate, which is the supporting structure of the cartilaginous endplate, showed typical degenerative changes following a 4-week compression (Fig. 5A). Irregular protrusions are visible on the rough bony endplate surface, and destruction of the original “concave lens” shape is accompanied by calcification, sclerosis and osteophyte formation to varying degrees. In addition, compared with the outer area of the endplate, the change in the central area is more pronounced, and the density and size of the pores decrease with destruction. After two to 4 weeks of traction intervention (LTT and HTT), pore morphology, density and diameter recovered significantly compared with Group A, and the LTT group was superior to the HTT group. In the statistics of pore number (density) (Fig. 5B), statistically significant differences were observed between the following group pairs: Group C vs Group D (*p* < 0.05), Group C vs Group E (*p* < 0.05) and Group D vs Group F (*p* < 0.05). No significant difference between Group B and Group A (*p* > 0.05) was detected. The pore diameter statistics showed that significant differences were detected between Group C vs Group E (*p* < 0.05), and Group D vs Group F (*p* < 0.05), consistent with the trend of pore density, and there was no significant difference in diameter between Group B and Group A (*p* > 0.05) (Fig. 5C). The LTT-4w group (Group F) displayed the strongest recovery of the four intervention groups.

### Micromechanical properties of intervertebral discs

**Fig. 6:**
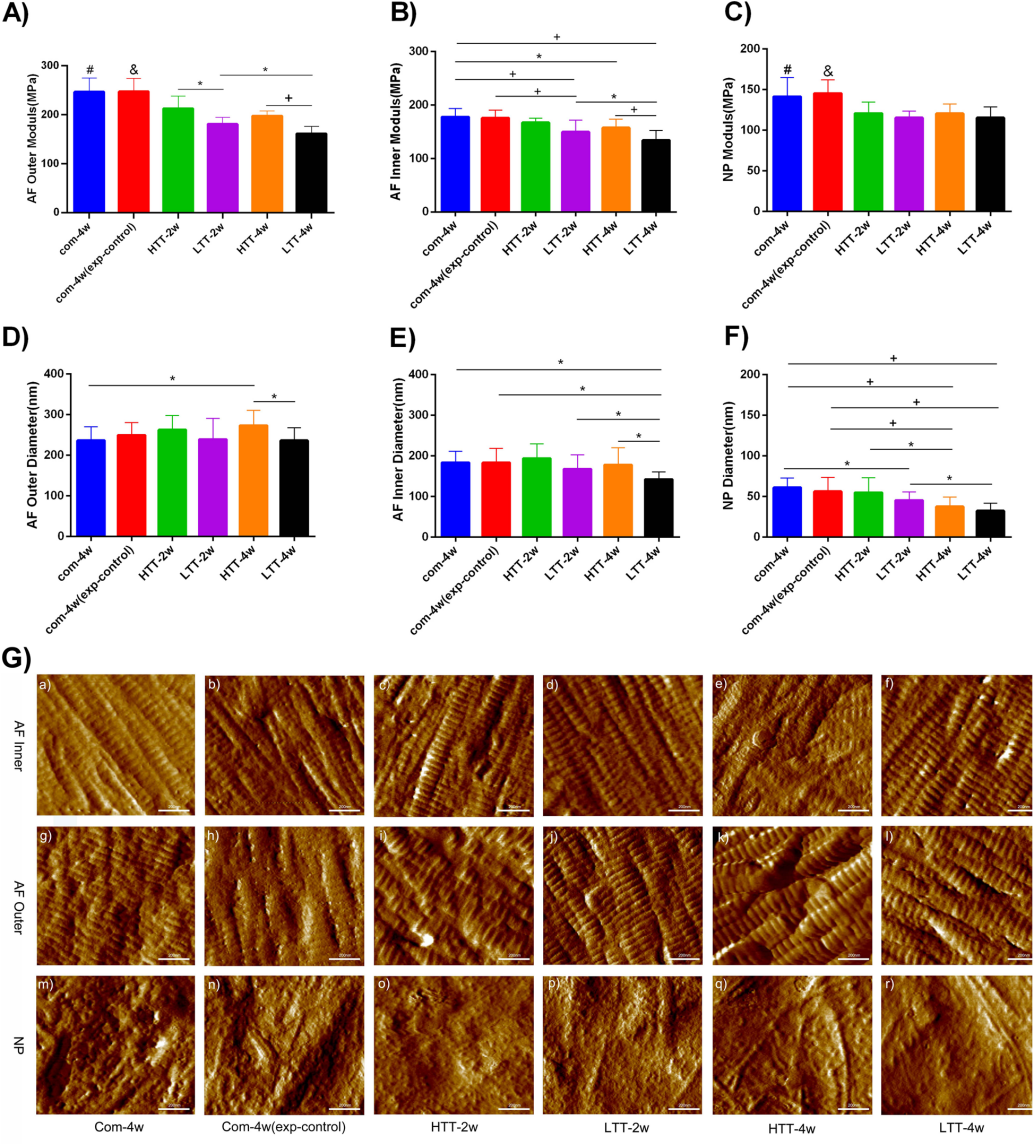
**A-F**) AFM was used to detect the elastic modulus and diameter of AF and NP collagen fibers. **G**) Representative AFM images of collagen fibrils in AF and NP. A-B) Nanoscale measurement of the elastic modulus of a single collagen fiber in AF. The diameter of AF is shown in Fig. D and E, and the elastic modulus and diameter of NP are shown in Fig. C and F: (#) represents significant difference compared with other groups except Group B (*p* < 0.05). (&) represents significant difference compared with other groups except Group A (*p* < 0.05). (*) indicates statistical significance between the two groups (*p* < 0.05). (+) indicates statistical significance between the two groups (*p* < 0.0001)**.**

The scanning diagrams of atomic force microscopy (AFM) from each region of AF and NP are shown in Fig. 2. Statistical analysis of the elastic modulus and diameter of collagen fibers is shown in Fig. 6. According to Fig. 6 A and B, under the same conditions, the elastic modulus of the outer collagen fiber is greater than that of the inner collagen fiber; that is, the outer AF is stiffer than the inner AF. The average elastic modulus of a collagen fiber in the AF of the intervention groups (Groups D to F, LTT group and HTT group) was significantly lower than that of the model group (Group A, *p* < 0.05). The LTT groups (D and F) were significantly better than the HTT groups (C and E; *p* < 0.05), and the LTT-4w group (Group F) had the best effect in terms of average elastic modulus. However, no significant difference was detected between the model group (Group A) and the experimental control group (Group B) (*p* > 0.05). The modulus trend of the inner AF was basically the same as that of the outer AF, except that no significant difference between the LTT -2w group (Group D) and the HTT -2w group (Group C) was detected (*p* > 0.05). Additionally, there was no significant difference  in the modulus between Group A and Group B. The results showed that the elastic modulus of AF in Group B was not significantly recovered after the removal of the external fixation device compared with the model group (Group A), while the intervention group (different intervention methods and cycles) showed obvious signs of recovery. The LTT groups were better than the HTT groups, and in particular the LTT-4w group was better than the 2w group.

Statistical analysis of the average diameter of collagen fibers in the inner and outer layers of AF is shown in Fig. 6 D-E. The figure shows that, consistent with the modulus trend, the average diameter of collagen fiber in the outer AF is larger than that in the inner AF; that is, the single collagen fiber in the outer AF is thicker than that in the inner AF. A possible explanation for this trend may be due to their higher elastic modulus. Fig. 6D shows that after traction intervention in the model group (Group A) with degeneration after 4 weeks of compression, no significant difference in the LTT-2w group or the HTT-2w group was observed. However, the extension of the traction period led to a larger average diameter in the HTT-4w group than in Group A (*p* < 0.05), indicating that excessive tension increases the tension of collagen fibers and compensatory thickening. In contrast, the LTT-4w group was statistically significant compared to the HTT-4w group (*p* < 0.05), indicating that moderately controllable traction tension can promote collagen fiber recovery. As shown in Fig. 6E, the diameter trend of the inner collagen fiber of the AF is basically consistent with that of the outer AF. Short-term traction intervention had little effect on collagen fibers in the AF, and the increase in traction period led to a decrease in the average diameter of collagen fibers. The LTT group was better than the HTT group, and the 4 week group was better than the 2 week group.

Fig. 6C-F shows that the modulus and diameter of the NPs are significantly smaller than those of the AF. Fig. 6C shows that after 2-4 weeks of intervention (LTT and HTT), the fiber modulus in both groups was significantly lower than that in the model group (*p* < 0.0001). However, no significant difference was detected in the influence of the four intervention groups (Groups C to F) on the collagen fibers of the NP (*p* > 0.05) or between Group A (model group) and Group B (experimental control group) (*p* > 0.05). The average diameter and modulus of collagen fibers in the NP were basically the same, except that the LTT group was better than the HTT group, and the four  week group was better than the two  week group (*p* < 0.05).

In conclusion, the elastic modulus and fiber diameter of collagen fibers in AF and NP varied with different intervention types and time cycles.

## Discussion

IVDD is initially a silent subclinical process, a continuous process in which the related properties of its substructures undergo gradual changes over several decades, resulting in structural destruction and functional impairment.

### The bony endplate microenvironment was better reconstructed by the LTT modality

Each part of the disc has its own unique mechanical properties. Damage to the mechanical properties of a substructure can lead to damage to the overall mechanical function of the disc. As a physical and mechanical barrier of intervertebral discs, BEPs have a unique three-dimensional network structure [5]. This network structure is pivotal in force perception and transfer [20], and the transport of various nutrients and metabolites is highly dependent on the size of network pores [21]. Therefore, the bony endplate must be porous to allow nutrients and metabolites to enter the disc [22, 23]. Simultaneously, it also acts as a biomechanical barrier, preventing the disc from directly exerting load on the vertebral body, which is important for the mechanical stability of the vertebral body and the nutrient metabolism of the disc [24–26]. Previous studies have shown that intervertebral disc nutrition is mainly provided by the convective diffusion of the endplate or the blood supply from the outer AF [25]. Therefore, the integrity of the cartilage endplate is essential for the stability of the entire vertebral body and the nutritional supply of the disc [27, 28]. Calcification of the cartilaginous endplate prevents nutrients from entering the intervertebral disc [29, 30] and may result in poor nutrient supply to the intervertebral disc [1, 31]. However, previous literature has confirmed that with increasing age or changes in the mechanical environment, the bony endplate gradually calcifies, and sclerosis occurs, which further affects the metabolism of nutrients in the cartilage endplate and intervertebral disc [7]. Zehra et al. found that mechanical stress was the decisive factor of endplate thickness and porosity, regardless of age and that endplate defects were closely related to intervertebral disc degeneration [23]. In previous and present studies, we found that progressive degeneration of the bony endplate was accompanied by gradual degeneration of the intervertebral disc, namely, with irregular protrusions, destruction, calcification, sclerosis, and osteophyte formation of varying degrees on the rough surface of the endplate [13]. After controlled traction, the recovery of the pore structure and density was obvious [13]. In this study, the pore density and diameter of the intervention group (LTT and HTT) were significantly improved compared with those of the model group (Group A). In particular, the LTT group was superior to the HTT group, and the 4-week group was superior to the 2-week group. It should be noted that compared with the model group (Group A), the control group (Group B) showed no significant improvement after spontaneous recovery after removal of the external coccygeal fixator. In conclusion, it is postulated that LTT can better promote the reconstruction of bony endplates at the same intervention time and amplitude based on the principle of biomechanical adaptability and is closely related to the intervention period.

### In addition to promoting the progressive repair of the BEP, the LTT modality also remodeled the elastic modulus and micro/nanostructure of the AF and NP

The “fibrillar anchoring system” of the annulus and endplate ensures the integrity and mechanical stability of the disc [32, 33]. Our previous studies have confirmed that disc degeneration occurs not only at the macro- and microscales but also at the nanoscale [15, 19]. AF is a highly organized and anisotropic tissue, and its elastic fibers are important to its overall mechanical properties and even the mechanical properties of the entire intervertebral disc [34, 35]. In addition, these studies also demonstrated structural and biomechanical changes in different regions of the AF that were loaded at the nanoscale [36]. Meakin et al. reported that during axial compression of intervertebral discs, NPs containing less glycosaminoglycan content were unable to bond well with the inner layer of the AF, resulting in excessive radial and axial stress on the outer layer of the AF and, consequently, in increased stiffness [37]. Based on this, the present study applied HTT and LTT to repair degenerative discs according to the principle of biomechanical adaptability. Continuous LTT and HTT improved the elastic modulus and structure of the AF and reduced the stiffness of collagen fibers, with the LTT-4w group producing a better effect than the LTT-2w group. This is consistent with the trend of repairing BEP with LTT. These results indicate that LTT, which is comparable to physiological load, can not only restore the height of the disc but also facilitate the reconstruction of the degenerative BEP, as well as the recovery of the lamellar structure of the AF and the improvement of its mechanical properties. As a tissue with nonlinear mechanical properties, the gradual recruitment of collagen fibers is distinguished from the toe region, heel region and highly rigid linear region, with the physiological state of the fibers being wavy or serrated [38]. When the fiber is stretched, it can be straightened gradually with minimal resistance, namely, pleated stretch (LTT). Once the fiber straightens (early in HTT), it begins to bear load. If it is stretched further (HTT), the fiber is destroyed. Possible mechanisms include fiber breakage or even fiber pulling, which was also confirmed by the progressive deterioration of the BEP in the HTT group. In other words, the layered stability of the AF was further damaged by HTT, and eventually degeneration was intensified. In addition, the influence trend of each intervention group on the modulus and diameter of NP collagen fibers was basically consistent with that of AF during this study. However, no significant difference in the effect of HTT and LTT on the elastic modulus of NP collagen fibers was detected. In addition, there was no significant difference between the intervention groups at 4 weeks and 2 weeks. This further indicates that NP, as a special biomechanical structure, withstands mainly compressive stress. Collagen fibers are arranged in an irregular network distribution, and there are often large gaps between collagen fibers. Generally, they do not bear a high tension load, but AF, which is neatly and closely arranged in layers anisotropically with collagen fibers, mainly bears tensile stress and torsional stress [39, 40]. In addition, our study confirmed that the outer annulus collagen fibers were thicker and stiffer than the inner annulus and NP fibers under the same conditions. Therefore, the effect of HTT and LTT on NP collagen fibers is far less sensitive than that of AF. This further suggests that the outer AF anchored to the BEP is actively reshaped with the reconstruction of the BEP under LTT intervention.

### Furthermore, LTT promoted the synthesis of extracellular matrix

The intervertebral disc is a tissue that can bear a high load, but too much or too little compressive or tensile stress will lead to catabolic reactions. In vitro studies have shown that stress within the physiological range promotes anabolism, while stress beyond this range promotes catabolism. For NP and AF, LTT tends to synthesize extracellular matrix, and HTT tends to decrease the expression of anabolic genes and increase the expression of catabolic and inflammatory factors [41–43]. Guehring et al. reported that, in comparison to compression loading, tension increased the expression of type 1 collagen, type 2 collagen, biglycan, and decorin genes. At the same time, the expression of BMP-2, MMP-1 and fibrinogen was downregulated because the reduction of matrix metal degrading enzymes, such as MMP-1, may lead to the reduction of apoptosis [44]. Proteoglycans are responsible for maintaining disc hydration and height. Loss of proteoglycans is one of the primary manifestations of intervertebral disc degeneration. Therefore, restoration of proteoglycan content is required for degenerative disc repair [45–47]. This study showed that in the case of disc space recovery, the recovery of proteoglycan content was significant in LTT compared with HTT, with the LTT-4w groups scoring better than the LTT-2w groups. It is worth noting that the experimental control group (Group B) recovered spontaneously after the removal of the external fixation device, and the proteoglycan content, disc height and signal recovered.

The intensity was basically consistent, and no significant increase was observed. This study further demonstrated that LTT, under physiological load, and HTT promoted anabolic responses, which further increased extracellular matrix synthesis. The mechanism of this result is associated with the active reconstruction of the BEP and the reopening of nutritional channels.

### The LTT modality is more conducive than HTT to the maintenance of a stable mechanical environment required for degenerative disc repair

The relationship between mechanical loading and disc health has long been studied [48, 49]. Mechanical factors have been demonstrated to significantly affect cellular function [50, 51]. It is well known that the shape and function of normal tissues depend on the ability of cells to sense and produce the appropriate tissue environment. At the tissue level, tension or “residual” stress regulates the balance of osmotic pressure. Similarly, at the disc level, residual stresses and strains exist even in the absence of loads due to the osmosis of proteoglycans. This study showed that compared with the model group, signs of disc recovery in the HTT and LTT groups were observed, with the recovery showing more prominently in the LTT group, further confirming that the abnormal mechanical environment after disc degeneration can be repaired by timely and controlled axial traction. However, intervertebral disc cells are sensitive to tensile strength, duration and cycle. AF cells were continuously and periodically pulled (traction) at physiological frequency (1 Hz) and low tension (1%) for more than 24 hours in vitro, which proved that the stability of the internal environment could be maintained [52, 53]. Nevertheless, with increased tension (5-18%) and duration (more than 4-6 hours), NP cells responded with increased catabolism (i.e., reduced proteoglycan and upregulation in the expression of NO, Cox2, and MMP3 genes) [53, 54]. Similarly, for rabbit NP cells, continuous periodic traction (10%, 0.5 Hz) significantly increased cell proliferation and collagen production within 1-2 days, but this anabolic effect disappeared after 4-8 days [55, 56]. The conversion of anabolism to catabolism by continuous periodic stretching may occur due to fatigue; that is, cells are unable to produce the energy needed for biosynthesis [42].

In this study, the LTT group showed significant signs of recovery, consistent with the above trend. From the perspective of clinical observation, we assume that part of the IVDD in the early origin of LBP clinical symptoms disappeared or improved after early axial traction, but the increase in traction load, duration of symptoms and progressive aggravation indicates that there is an optimal range of parameters (duration and intensity) for axial traction, beyond which symptoms reappear and even aggravate disc degeneration.

However, as with all animal studies, the limitations of this study are that the rat coccyx model cannot truly simulate the biochemical, molecular and biomechanical properties of human IVD. While not completely equal to the human IVD changes, the rat model is the currently accepted model and was easier to build and replicate than alternatives. In addition, in order to be different from other caudal puncture models and better simulate the degeneration state of human IVD mechanics, male SD rats aged 6 months (approximately 18 years old) [57] were selected to more realistically simulate the development and degeneration of human IVD, both in terms of social maturity phase and skeletal maturity.

## Conclusions

This study reconfirmed that the low-tension traction (LTT) modality blocked the cascade of disc degeneration to some extent. The LTT model improved the synthesis of the extracellular matrix of the degenerative disc while reconstructing the bony endplate. Compared with the high-tension traction (HTT) model, the micro/nanostructure and mechanical properties of the annulus fibrosus and nucleus pulposus were effectively improved, and the stability of the microenvironment of degenerative intervertebral discs was maintained, providing a better guarantee for the regeneration, repair and reconstruction of degenerative intervertebral discs.

## Data Availability

The datasets used and/or analyzed during the current study are available from the corresponding author on reasonable request.
